# Direct Parametric Maps Estimation from Dynamic PET Data: An Iterated Conditional Modes Approach

**DOI:** 10.1155/2018/5942873

**Published:** 2018-07-08

**Authors:** Michele Scipioni, Assuero Giorgetti, Daniele Della Latta, Sabrina Fucci, Vincenzo Positano, Luigi Landini, Maria Filomena Santarelli

**Affiliations:** ^1^Dipartimento di Ingegneria dell'Informazione, University of Pisa, Pisa, Italy; ^2^Fondazione Toscana G. Monasterio, Via G. Moruzzi 1, 56124 Pisa, Italy; ^3^CNR Institute of Clinical Physiology, Via G. Moruzzi 1, 56124 Pisa, Italy

## Abstract

We propose and test a novel approach for direct parametric image reconstruction of dynamic PET data. We present a theoretical description of the problem of PET direct parametric maps estimation as an inference problem, from a probabilistic point of view, and we derive a simple iterative algorithm, based on the Iterated Conditional Mode (ICM) framework, which exploits the simplicity of a two-step optimization and the efficiency of an analytic method for estimating kinetic parameters from a nonlinear compartmental model. The resulting method is general enough to be flexible to an arbitrary choice of the kinetic model, and unlike many other solutions, it is capable to deal with nonlinear compartmental models without the need for linearization. We tested its performance on a two-tissue compartment model, including an analytical solution to the kinetic parameters evaluation, based on an auxiliary parameter set, with the aim of reducing computation errors and approximations. The new method is tested on simulated and clinical data. Simulation analysis led to the conclusion that the proposed algorithm gives a good estimation of the kinetic parameters in any noise condition. Furthermore, the application of the proposed method to clinical data gave promising results for further studies.

## 1. Introduction

Dynamic positron emission tomography (PET) consists of the acquisition of a sequence of 3D images over time to follow the uptake and washout of an injected radiotracer in the imaged object. Hence, dynamic PET provides additional temporal information about tracer kinetics compared to static PET. The pharmacokinetic analysis allows, then, to estimate biologically relevant kinetic parameters from the measured concentration in tissue over time, that is, the tissue's time activity curve (TAC). These parameters can provide a greater insight into the diagnosis of several diseases, helping clinicians in differentiating among different kind of lesions, and can assist during the follow-up of treatment response in a more specific way than the simple evaluation of the standard uptake value at a single time point [1].

There are two main approaches to characterize tracer kinetics from dynamic PET data: region-of-interest (ROI) kinetic modeling and parametric imaging [2, 3]. The ROI-based approach fits a kinetic model to the average TAC of a selected ROI. On the contrary, parametric imaging aims at estimating kinetic parameters for every voxel, thus providing a representation of their spatial distribution as parametric maps.

Parametric images have been found useful to enhance characterization of the regional heterogeneity. However, this technique is more computationally demanding, and more sensitive to noise in dynamic PET data than the ROI-based kinetic modeling [4]. The main difficulties related to estimating these maps are that a single voxel's TAC is noisier than the average TAC used for ROI-based modeling and that the voxel-wise estimate needs to be performed in a sequential way, without the possibility to exploit information about spatial proximity as regularization constraint during the fitting of complex kinetic models. This can lead to different optimal parameter sets even for voxels belonging to the same tissue and supposedly sharing the same kinetic behavior; this produces potentially noisy and biased parametric maps. There is some research about the effects of spatial regularization on kinetic parameter estimation [5], but this approach is not commonly used because it requires huge parallelization power to process all the voxel in the 3D volume at the same time and because it could easily lead to biased maps, if the spatial smoothing is too strong.

The standard approach to process dynamic PET scans (4D PET) starts from the independent reconstructions of series PET images, acquired in a certain number of consecutive time frames. This can potentially lead to low count statistics, according to the chosen time scheme, which determines the length of each time frame [6]. Due to the ill-conditioning of the tomographic problem, iterative reconstruction algorithms, such as the maximum likelihood expectation maximization (MLEM) method, are a common choice, even if the filtered backprojection (FBP) method is sometimes preferred. The pharmacokinetic modeling is typically applied later, and thus, it is sensitive to noise in the previously reconstructed PET images. An efficient and unbiased estimate of the kinetic parameters of the chosen model requires properly taking into account the noise distribution of the reconstructed activity images [7] or a parametric images denoising by means of a spatial regularization [5]. However, for the frequently used iterative image reconstruction algorithms, this noise distribution is rarely known precisely and very hard to model with a known mathematical form because it is related to the number of iterations employed and other features of the reconstruction algorithm [8–10].

Postprocessing techniques such as filtering or ROI-based analysis (that, e.g., clusters voxel with similar kinetics) exist. However, all postreconstruction modeling approaches are fundamentally limited as they operate only indirectly on the reconstructed images so that the noise statistics cannot be accurately modeled. Inaccurate modeling of noise statistics may result in unreliable estimation of voxel-wise kinetic parameters, particularly if there are high levels of noise in the acquired data.

Given these considerations, the growing interest in fully 4D image reconstruction techniques can be easily understood. 4D reconstruction methods try to address the problems of noise characterization and limited counts in dynamic emission tomography by incorporating a theoretical model of the temporal behavior of the radiotracer directly into the image reconstruction algorithm [7, 11, 12]. This approach allows for adding physiologically meaningful constraints to the reconstruction process itself, and it provides reliable estimations of voxel-wise kinetic parameters directly from raw data by exploiting the same mathematical models normally used on a postreconstruction basis [2, 3].

It is well known that PET projection data can be described as independent Poisson realizations. Directly estimating kinetic parameters from sinogram data facilitates accurate modeling of noise distribution which follows Poisson statistics and gives an accurate compensation of the noise propagation, from projection measurement to kinetic fitting. This peculiar property has been shown [13–15] to provide a better bias-variance characteristic with respect to the conventional indirect approaches, both using linear and nonlinear kinetic models.

In this work, we propose a theoretical description of the problem of PET direct parametric maps estimation as an inference problem, from a probabilistic point of view. This assumption allows us to derive a simple two-step iterative optimization algorithm, based on the Iterated Conditional Mode (ICM) framework [16]. The resulting method is general enough to be flexible to an arbitrary choice of the kinetic model, and unlike many other solutions [17–19], it is natively capable to deal with nonlinear compartmental models without the need for linearization. For the testing purpose, we chose to couple the proposed optimization method with a two-tissue compartment model [2, 3], including an analytical solution to the kinetic parameters evaluation problem, based on an auxiliary parameter set, with the aim of reducing computational errors and approximations. The new method is tested on simulated and clinical data.

## 2. Materials and Methods

### 2.1. PET Data Model

Let us approximate the radiotracer activity within the region-of-interest of the subject's body using a set of point sources *x*={*x*_*j*_},*j* ∈ {1,…, *J*}, displaced on a regular voxel grid. Since emission events in the same voxel are not time correlated, their rate can be described as a Poisson distribution, with the expected value *x*_*j*_. The geometry of the acquisition system and attenuation determines the probability *p*_*ij*_ of a photon emitted by voxel *j* being detected by line of response (LOR) *i*. From the sum property of Poisson distribution, counts *y*_*i*_ recorded in *i* are, again, Poisson distributed, with the expected value ∑_*j*_ *p*_*ij*_ *x*_*j*_, and measurements in each detector bin *i* are independent, conditionally to activity [20, 21]. Keeping in mind that in dynamic PET imaging both activity image and sinogram counts are functions of time, we can express the probability to observe *y*={*y*_*i*_}, *i* ∈ {1,…, *I*} given *x* as

(1) $$\begin{array}{c} p\left(y\;|\;x\right)=\overset{I}{\underset{i=1}{\prod }}\text{Poisson}\left(\underset{j}{\sum }p_{ij}x_{j},y_{i}\right). \end{array}$$

The aim of direct parametric PET reconstruction is to generate parametric images {*θ* ^*p*^}, *p*=1,…, *P*, with *P* number of model parameters, directly from the measured raw dynamic data and to use them to guide the image reconstruction process [7, 12, 13, 15, 22, 23]. If we define a vector *θ*_*j*_ ∈ *ℝ* ^*P*^, which contains the *P* parameters related to voxel *j*, and we identify with *f*(*t*, *θ*_*j*_) a generic kinetic model, which provides a theoretical representation of the time activity curve (TAC) for voxel *j* over time, the relationship between the model and the reconstructed voxel TAC can be expressed as follows:

(2) $$\begin{array}{c} x_{jm}\left(\theta_{j}\right)=f\left(t_{m},\theta_{j}\right)+\varepsilon . \end{array}$$

Irrespectively of the chosen kinetic model *f*(·), (2) encodes the assumption that voxel intensities can be modeled as noisy realizations of a hidden dynamic process, and each voxel's TAC can be parameterized using a kinetic model, defined on a set of *P* parameters. Given (2), we decided to model the uncertainty *ε* using a probability distribution: for each time point *t*_*m*_, the corresponding value of *x*_*jm*_ has a Gaussian distribution with a mean equal to value *f*(*t*_*m*_, *θ*_*j*_) of the model curve and standard deviation *σ*. Thus, we have

(3) $$\begin{array}{c} p\left(x_{j}\;|\;\theta_{j}\right)=\overset{M}{\underset{m=1}{\prod }}N\left(x_{jm}\;|\;f\left(t_{m},\theta_{j}\right),\sigma \right)\propto \;\overset{M}{\underset{m=1}{\prod }}\exp\left(\frac{-1}{2\sigma^{2}}{\left\|x_{jm}-f\left(t_{m},\theta_{j}\right)\right\|}^{2}\right). \end{array}$$

Given (1) and (2), we can explicit the connection between measured sinogram counts and model parameters through the following reinterpretation of the standard affine model of PET data:

(4) $$\begin{array}{c} y_{im}\left(\theta \right)=\overset{J}{\underset{j=1}{\sum }}p_{ij}x_{jm}\left(\theta_{j}\right)+r_{im}, \end{array}$$

where *r*_*im*_ is the estimate of random and scattered events occurring in the same LOR *i*, during time frame *m*. This allows us to directly express the Poisson likelihood in (1) as a *p*(*y* |  *θ*), function of model parameters *θ*, instead of activity values *x*.

### 2.2. ICM-Based 4D Reconstruction

Instead of directly deriving an optimization algorithm to maximize the likelihood *p*(*y* |  *θ*), as done in other works about direct PET maps estimation [7, 12, 13, 15, 22, 23], we made use of the Iterated Conditional Modes (ICM) framework, introduced by Besag [16]. ICM consists of splitting the optimization problem by maximizing, in turn, each conditional probability, given the provisional estimates of the other variable: this procedure defines a single cycle of an iterative algorithm to estimate all the variables.

Considering the Poisson likelihood in (1) and the Gaussian likelihood in (3), we alternatively estimate (i) the parameters of the kinetic model with the highest probability given the activity and (ii) the activity with highest probability given the parameters and the PET projection data.

- Given the provisional estimate of activity $\hat{x}$, we can adopt a maximum likelihood approach to maximize the logarithm of the likelihood function, which has arisen from the assumption of Gaussian noise distribution in (3). It is easy to see how maximizing the Gaussian log-likelihood is equivalent to minimizing the *sum of squares error function* between model and voxel's TAC:

(5) θ^jn+1=argminθj∑m=1Mx^jmn−ftm,θj2,

which can be done using any conventional nonlinear least-squares (NLS) method (e.g., the Levenberg–Marquardt algorithm [23]).

(ii) Given the updated parametric maps (i.e., parameter vector *θ*_*j*_ for each voxel of the volume of interest), we now want to maximize *p*(*y* |  *x*, *θ*) of (1) incorporating the result from the previous step. Inspired by the approach proposed by Wang and Qi [24, 25] (in their optimization transfer expectation maximization (OT-EM) algorithm for PET direct reconstruction) for transferring optimization of the Poisson likelihood from the image to parameters domain, we chose to maximize this conditional *pdf* as follows:

(6) $$\begin{array}{c} {\hat{x}}_{jm}^{\left(n+1\right)}=\frac{{\bar{x}}_{jm}^{\left(n+1\right)}}{\overset{I}{\underset{i=1}{\sum }}p_{ij}}\overset{I}{\underset{i=1}{\sum }}p_{ij}\frac{y_{im}}{\overset{J}{\underset{j^{\prime }=1}{\sum }}p_{ij^{\prime }}{\bar{x}}_{jm}^{\left(n+1\right)}}, \end{array}$$

where x¯jmn+1=ftm,θ^jn+1 is the mean value of activity in voxel *j* at time frame *m*, given the updated estimate of model parameters performed in the previous step.

For the rest of this work, we will denote this concurrent optimization algorithm as Iterated Conditional Modes based Expectation Maximization (ICM-EM) reconstruction.

### 2.3. Kinetic Modeling with a Two-Tissue Compartmental Model

The proposed inference framework was described in the previous section as independent of the choice of the kinetic model. However, we now need a proper expression for the theoretical model *f*(*t*_*m*_, *θ*_*j*_) in (2) and (5), able to describe the behavior of the tracer in tissue. While linear models are often the preferred choice because of their computational efficiency and robustness, nonlinear ones could provide a greater insight into the biochemical properties of the various tissues [2, 3]. Most of these nonlinear models are based on compartments to describe the behavior of the tracer: each compartment identifies either a distinct physical location or a different chemical state of the tracer. The unknown parameters of the model are constant transfer rates, which describe the flow of the tracer among different compartments.

Following the compartmental model theory [3], the total tracer concentration in a tissue region can be modeled as follows:

(7) $$\begin{array}{c} {\hat{C}}_{T}\left(t\right)=\left(1-f_{v}\right)\left[C_{p}\left(t\right)\;⊗\;\overset{C}{\underset{c=1}{\sum }}c_{c}\left(t\right)\right]e^{-d_{k}t}+f_{v}C_{\text{wb}}\left(t\right), \end{array}$$

where *f*_v_ is the fractional volume of blood in tissue, *d*_*k*_ is the radioactive decay constant of the chosen tracer (e.g., for [18F]FDG, *d*_*k*_=ln(2)/109.8min^−1^), *C*_p_ is the measured tracer concentration in plasma, *C*_wb_ is the concentration in the whole blood, and **c**_c_(*t*) represents the expected impulse response function (IRF) in the *c*th compartment. The relationship between compartment concentrations is usually described by a set of ordinary differential equations (ODEs):

(8) $$\begin{array}{c} \frac{d}{dt}c=\mathrm{Kc}+\mathrm{Lu}, \end{array}$$

where **c**=[**c**_1_,…,**c**_C_]^*T*^, **K** and **L** are the model constant parameters matrices, and **u** denotes the system's input function. In the common case of a two-tissue compartment model, we have

(9) $$\begin{array}{c} c=\left[\begin{array}{c} C_{f}\\ C_{b} \end{array}\right],\\ u=C_{p},\\ K=\left[\begin{array}{cc} -\left(k_{2}+k_{3}\right) & k_{4}\\ k_{3} & -k_{4} \end{array}\right],\\ L=\left[\begin{array}{c} K_{1}\\ 0 \end{array}\right], \end{array}$$

where with *C*_f_ and *C*_b_ we distinguish between the concentration of the free and bound compartments of a two-tissue model. The system of ODE in (8) can be solved analytically in case of an impulsive input **u**, and the impulse response function (IRF) is

(10) $$\begin{array}{c} c\left(t_{m}\right)=\frac{K_{1}}{\left(\beta_{2}-\beta_{1}\right)}\left[\begin{array}{cc} k_{4}-\beta_{1} & \beta_{2}-k_{4}\\ k_{3} & -k_{3} \end{array}\right]\left[\begin{array}{c} e^{-\beta_{1}t_{m}}\\ e^{-\beta_{2}t_{m}} \end{array}\right], \end{array}$$

where $\beta_{1,2}=1/2\left[\left(k_{2}+k_{3}+k_{4}\right)\pm \sqrt{{\left(k_{2}+k_{3}+k_{4}\right)}^{2}-4k_{2}k_{4}}\right]$. As shown by Gunn et al. [3], this solution can be further simplified introducing a set of auxiliary parameters Φ_aux_=[*f*_v_, *α*_1_, *α*_2_, *β*_1_, *β*_2_]^*T*^:

(11) $$\begin{array}{c} \alpha_{1}=K_{1}\frac{k_{3}+k_{4}-\beta_{1}}{\beta_{2}-\beta_{1}},\\ \alpha_{2}=K_{1}\frac{\beta_{2}-k_{3}-k_{4}}{\beta_{2}-\beta_{1}}, \end{array}$$

so that we can express the tissue IRF as a sum of two exponential functions:

(12) $$\begin{array}{c} c\left(t_{m}\right)=\alpha_{1}e^{-\beta_{1}t_{m}}+\alpha_{2}e^{-\beta_{2}t_{m}}=\overset{2}{\underset{c=1}{\sum }}\alpha_{c}e^{-\beta_{c}t_{m}}. \end{array}$$

### 2.4. Modeling of the Input Function

Once we have the tissue IRF, from system theory we know that the output of our dynamic system with respect to a generic input can be computed by convolving (12) with the system input function, *C*_p_. Combining (7) with (12), we obtain

(13) $$\begin{array}{c} {\hat{C}}_{T}\left(t\right)=\left(1-f_{v}\right)\left[C_{p}\left(t\right)\;⊗\;\overset{2}{\underset{c=1}{\sum }}\alpha_{c}e^{-\beta_{c}t}\right]e^{-d_{k}t}+f_{v}C_{\text{wb}}\left(t\right). \end{array}$$

This means that tracer kinetic modeling with PET requires to measure the tracer time activity curves in both plasma and tissue to estimate physiological parameters.

In the present work, we decided to adopt a theoretical representation also for the input function, using a combination of exponential terms, that for [18F]FDG tracer is given by the following equation [26]:

(14) $$\begin{array}{c} {\hat{C}}_{p}\left(t_{m}\right)=\left\{\begin{array}{cc} \left[A_{1}\left(t_{m}-t_{0}\right)-A_{2}-A_{3}\right]e^{-\lambda_{1}\left(t_{m}-t_{0}\right)}+A_{2}e^{-\lambda_{2}\left(t_{m}-t_{0}\right)}+A_{3}e^{-\lambda_{3}{\left(t_{m}-t_{0}\right)}_{}}, & \text{for }t_{m}\geq t_{0},\\ 0, & \text{for }t_{m}<t_{0}. \end{array}\right. \end{array}$$

### 2.5. Analytical Tissue Compartment Modeling

The convolution operation in (13) is usually performed numerically, for each voxel in our volume, and this operation can be computationally and time expensive, given the size of the 4D volumes we are dealing with. It is interesting to note how this operation can be computed more efficiently when discretization is avoided [27].

Using Feng's input function model (14), with an amplitude vector *A*=[*A*_1_, *A*_2_, *A*_3_]^*T*^, and inverse time constant vector *λ*=[*λ*_1_, *λ*_2_, *λ*_3_]^*T*^, we can derive an analytical solution to the convolution operation, so to avoid entirely the numerical convolution and temporal sampling of the input function. Expanding the convolution operator in the output function (13), substituting the input function model (14), and performing a series of algebraic manipulations, the output function can be expressed analytically as follows:

(15) $$\begin{array}{c} {\hat{C}}_{T}\left(t_{m}\right)=\overset{2}{\underset{c=1}{\sum }}\frac{A_{1}t_{m}\alpha_{c}}{\left(\beta_{c}-\lambda_{1}\right)}e^{-\lambda_{1}t_{m}}+\overset{2}{\underset{c=1}{\sum }}\overset{3}{\underset{j=1}{\sum }}\frac{{\hat{A}}_{j}t_{m}\alpha_{c}}{\left(\beta_{c}-\lambda_{j}\right)}\left(e^{-\lambda_{j}t_{m}}-e^{-\beta_{c}t_{m}}\right), \end{array}$$

where $\hat{A}=\left[-A_{2}-A_{3}-\left(A_{1}/\left(\beta_{c}-\lambda_{1}\right)\right),-A_{2},-A_{3}\right]$.

Adding to equation (15), the correction factors for radioactive decay and blood fraction in tissue, as in (7), we obtain the final version of our two-tissue analytic compartment model:

(16) $$\begin{array}{c} {\hat{C}}_{T}\left(t_{m}\right)=\left(1-f_{v}\right)\left[\overset{2}{\underset{c=1}{\sum }}\frac{A_{1}t_{m}\alpha_{c}}{\left(\beta_{c}-\lambda_{1}\right)}e^{-\lambda_{1}t}+\overset{2}{\underset{c=1}{\sum }}\overset{3}{\underset{j=1}{\sum }}\frac{{\hat{A}}_{j}t_{m}\alpha_{c}}{\left(\beta_{c}-\lambda_{j}\right)}\left(e^{-\lambda_{j}t_{m}}-e^{-\beta_{c}t_{m}}\right)\right]e^{-d_{k}t_{m}}+f_{v}{\hat{C}}_{p}\left(t_{m}\right). \end{array}$$

### 2.6. Algorithm Implementation

The ICM-EM algorithm here proposed was implemented in Matlab (The MathWorks, Inc., Natick, Massachusetts, United States).

For the first step of the algorithm (5), we used the *lsqcurvefit* function, provided by Matlab with the Optimization Toolbox, for nonlinear least-squares fitting with a Levenberg–Marquardt algorithm [23]: we chose to constrain the search space for the kinetic constants between 0 and 10, and for the *f*_v_ parameter, between 0 and 1. Default stopping conditions were used.

We, then, used the *Image Reconstruction Toolbox* (IRT), provided by Fessler [28], to implement the second step (6) of updating the activity estimate given the new estimate of parametric maps and sinogram data. We exploited this tool for system matrix generation, based on a square pixel basis and strip-integral detector model, and we applied the required modification to the IRT's MLEM algorithm in order to correctly implement (6) for 2D single-slice dynamic PET data. Before reconstructing, images were initialized by setting all voxels inside the field of view (FOV) to 1.0 and all *k* parameters to 0.1.

### 2.7. Simulation

A numerical PET phantom was created starting from the Zubal brain phantom [29] image of 111 × 111 points, which includes white matter and gray matter. The field of view was 70 cm. We simulated an ideal measurement system with ideal detectors with equal sensitivity, efficiency, and detection cross section.

Dynamic data were generated by applying on each pixel the TAC curve that follows the kinetic parameter behavior according to the two-compartment model. The kinetic parameter values used for the simulation of both gray matter and white matter are shown in Table 1. The blood input function was modeled according to (14), with the following parameter values: *A*_1_=10, *A*_2_=0.5, and *A*_3_=2, and *λ*_1_=0.5, *λ*_2_=0.05, and *λ*_3_=0.005.

**Figure 1:**
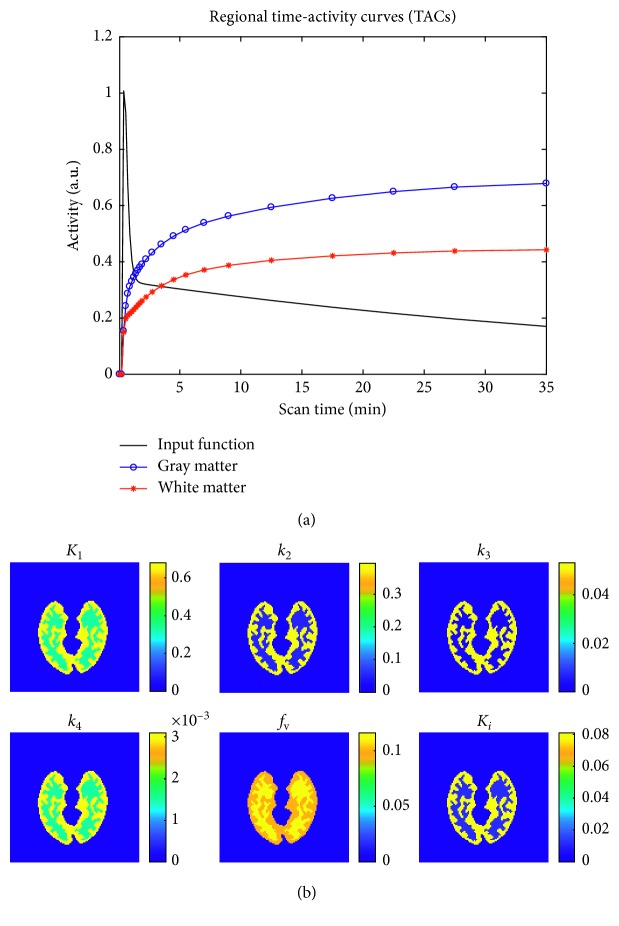
Simulation starting data: (a) TACs relevant to input function, gray matter, and white matter; (b) parametric maps.

In Figure 1, the TACs relevant to input function, gray matter, and white matter are shown; in Figure 1(b), the geometric arrangement in the brain of the kinetic parameters are shown.

A time series of Poisson-distributed sinograms was generated by projecting the simulated dynamic activity. Each sinogram consisted of 367 × 315 points (number of bins × number of angles): we denote as *m*Sin, the mean value of each sinogram's counts.

A simplified model of attenuation was applied: the attenuation map was modeled as a 111 × 111 matrix, with the attenuation coefficient equal to 0.098 cm^−1^ [10]. The scattering effect was implemented according to the single-scatter simulation (SSS) algorithm [30], while the random coincidences were modeled as a uniform value. Both these effects were proportional to the mean count rate of the simulated sinograms, *m*Sin; several noise levels (from 10% to 80% of *m*Sin) were simulated to evaluate their influence on the kinetic parameters estimation. Scattering and random counts effect was applied to each sinogram according to (4). Also, the radioactive decay effect was included, considering the [18F]FDG half-life of 109.8 minutes.

The resulting dynamic projections were then considered as raw input for the ICM-EM algorithm and the parametric maps, one for each kinetic parameter, were generated. The number of iterations for the EM part of the algorithm (6) was fixed to 2, while the iterations number of the whole alternate direct optimization (i.e., *n* in (5) and (6)) ranged from 2 to 50, in order to evaluate which is the optimal number of iterations to apply, also in presence of noise.

The simulation described above was repeated 20 times, in a Monte Carlo framework, giving a much more comprehensive view of the results.

All the resulting maps were quantitatively analyzed by calculating the mean squared error (MSE) index, which gives information about the goodness of the estimation. The MSE index allows comparing the estimated kinetic maps with the relevant true values given as input to the simulation; it is computed as follows:

(17) $$\begin{array}{c} \text{MSE}=\frac{1}{J}\overset{J}{\underset{j=1}{\sum }}{\left(k_{j}-{\hat{k}}_{j}\right)}^{2}, \end{array}$$

where *J* is the number of pixels in the map, *k*_*j*_ is the true value of the *j*th pixel in the simulated map, and ${\hat{k}}_{j}$ is the value of the *j*th pixel in the estimated map. The MSE was evaluated for different noisy conditions and number of iterations.

The normalized log-likelihood norm*L*_*n*_ was calculated at each direct iteration *n*, to evaluate the convergence rate of the ICM-EM algorithm. It is given by

(18) $$\begin{array}{c} \mathrm{norm}L_{n}=\frac{L_{n}-L_{\min}}{L_{\max}-L_{\min}}, \end{array}$$

where *L*_max_ and *L*_min_ are, respectively, the maximum and minimum log-likelihood values.

### 2.8. Clinical Data

Six [18F]FDG dynamic PET data sets were acquired by using a PET/CT Discovery RX VCT 64-slice (GE Healthcare, Milwaukee, WI) scanner, on subjects without cerebral pathologies. The study complied with the Declaration of Helsinki. All subjects gave written informed consent to the protocol. The project was approved by the institutional ethics committee.

The acquisition protocol started few seconds after the injection of [18F]FDG (5 MBq/kg body weight). The scan protocol consisted of 24 frames (12 × 10 s, 2 × 30 s, 3 × 60 s, 2 × 120 s, 4 × 300 s,  and 1 × 600 s for a total of 40 min) of 3D data (47 slices each one). The single sinogram slice was of 367 × 315 values, for a field of view (FOV) of 70 cm.

For each subject's data, the input function was derived by properly selecting a region-of-interest from a vascular region (i.e., carotid) on a preliminary reconstruction of the dynamic series with an ordered subset EM (OSEM) algorithm (21 subsets; 3 iterations). This experimental input function was then fitted using the Feng model in (14), using the NLS fitting algorithm.

The acquired raw data, attenuation maps, random and scatter correction matrices, and the input functions were transferred from the scanner to an external workstation, to generate kinetic maps. The kinetic behavior of [18F]FDG was modeled using a two-tissue compartment. The kinetic parameters estimated were *K*_1_,…, *k*_4_, *f*_v_, and the uptake rate of [18F]FDG, *K*_*i*_, was *K*_*i*_=*K*_1_ *k*_3_/(*k*_2_+*k*_3_). The parametric maps were reconstructed using the same proposed ICM-EM direct reconstruction algorithm used in the simulation.

For each subject's data, PET images were generated by applying the ICM-EM algorithm using 10 iterations, and from these images, two anatomical regions of interest (ROIs) were selected, covering, respectively, gray and white matter region of the brain. Then, the kinetic parameter values relevant to the two different ROIs were evaluated for several different numbers of direct reconstruction iterations.

## 3. Results

### 3.1. Simulated Data

**Figure 2:**
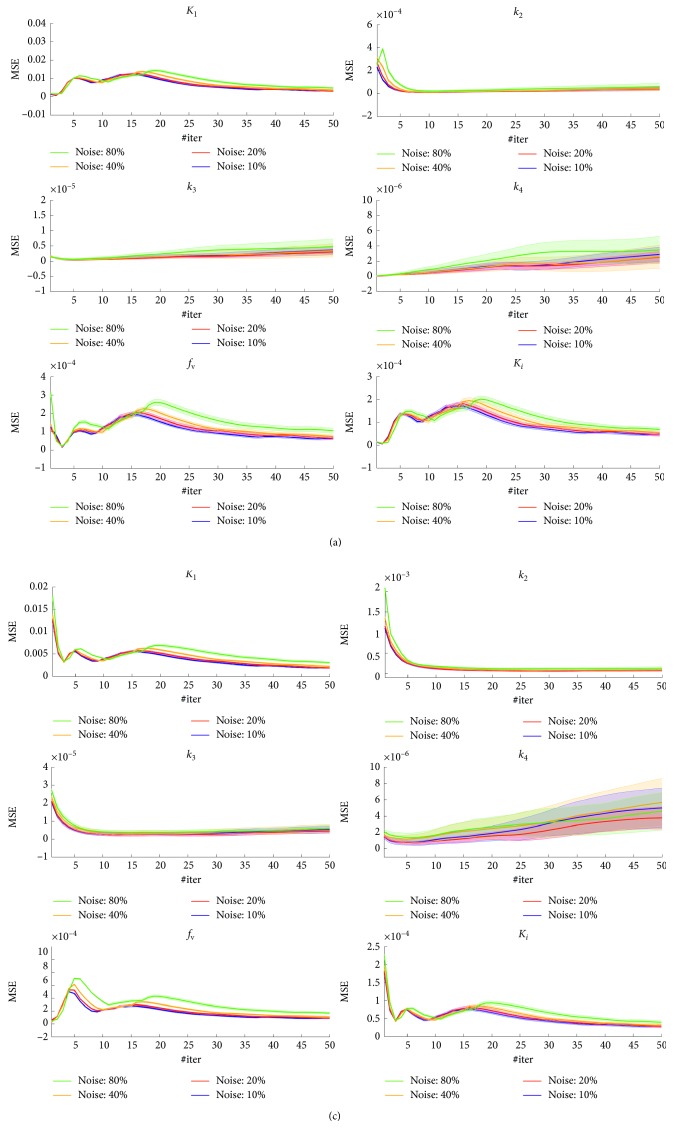
MSE evaluated on the parametric maps *K*_1_,…, *k*_4_, *f*_v_, and *K*_*i*_ as function of the direct reconstruction iterations number (#iter), in four different noisy conditions: (a) gray matter; (b) white matter.

In Figure 2, it is shown the mean and standard deviation of the MSE index, evaluated on the parametric maps *K*_1_,…, *k*_4_, *f*_v_, and *K*_*i*_ by ICM-EM algorithms as a function of the reconstruction iterations number, #iter. Results are relevant to four different noisy conditions: total  noise=[10%, 20%, 40%, 80%] of the dynamic data sinograms mean value, *m*Sin.

**Figure 3:**
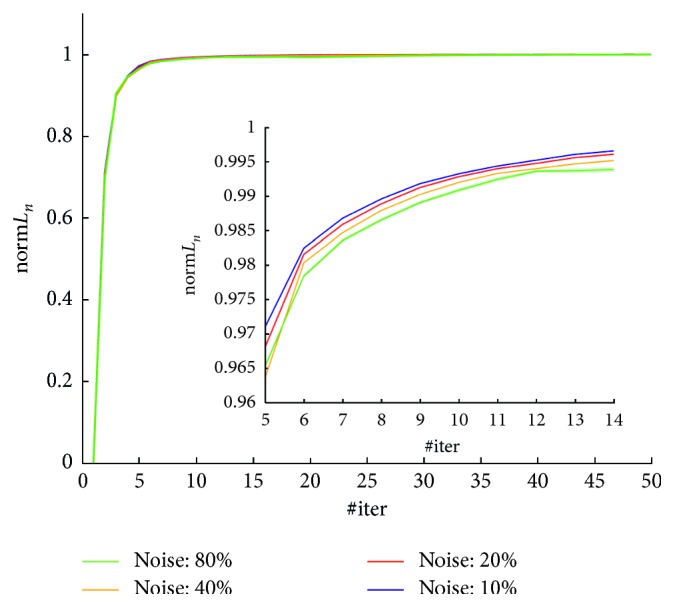
Normalized log-likelihood norm*L*_*n*_, of the ICM-EM algorithm, for four different noisy conditions. Inner box: zoomed version of the graph.

Results obtained by evaluating the normalized log-likelihood norm*L*_*n*_ are shown in Figure 3. Four different noise conditions are shown from 10% to 80% of *m*Sin. In Figure 3 also zooms of the graphs are shown, to better distinguish the behavior of the *n* in different noisy conditions.

**Figure 4:**
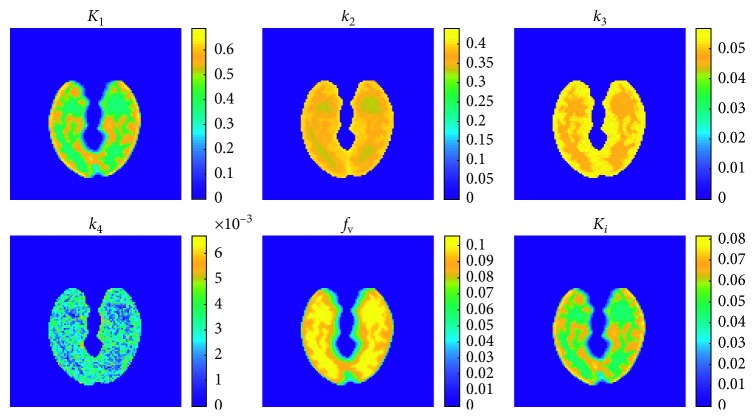
Parametric maps obtained from simulated data with 40% of noise and reconstructed with ten direct iterations.

Figure 4 shows an example of the resulting parametric maps obtained from simulated data with 40% of noise and reconstructed with 10 direct iterations.

**Figure 5:**
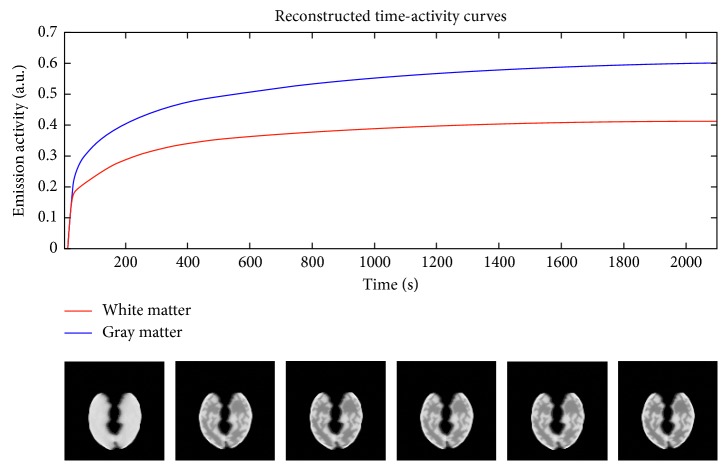
Dynamic reconstructed images obtained from simulated data with 40% of noise and reconstructed with ten direct iterations: (a) TACs reconstructed from the images; (b) reconstructed temporal images.

Figure 5 shows the dynamic reconstructed images, obtained with the same conditions as in Figure 4. Figure 5 shows the TACs reconstructed from the images obtained applying the ICM-EM algorithm; they are relevant to a white and a gray matter region. Figure 5 shows image frames relevant to six different time intervals.

### 3.2. Clinical Data

**Figure 6:**
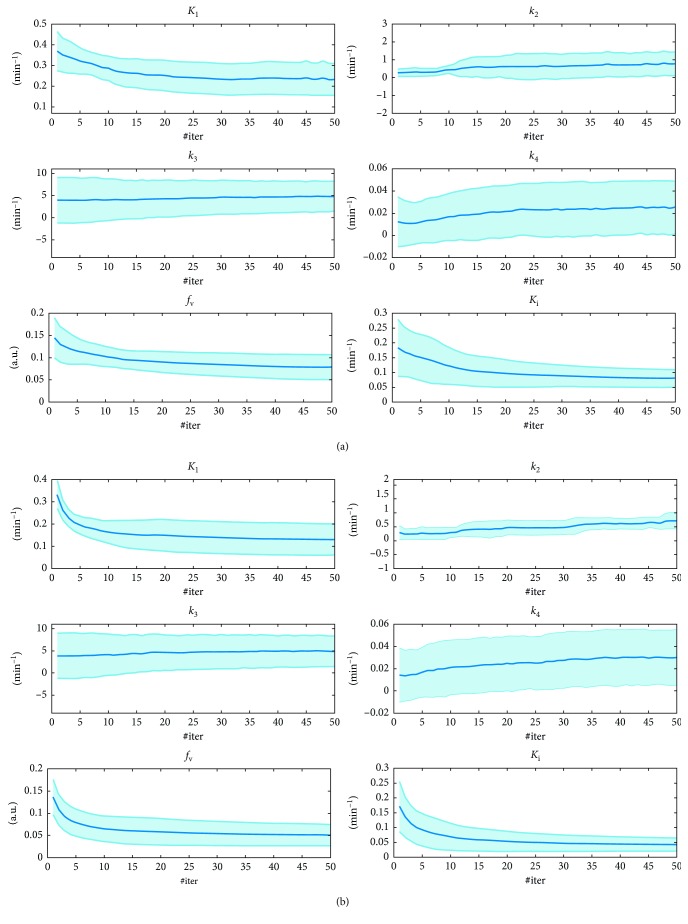
Estimated kinetic parameters *K*_1_,…, *k*_4_, *f*_v_, and *K*_*i*_, from two ROIs on clinical data, as function of the direct reconstruction iterations number (#iter): (a) gray matter; (b) white matter.

Figure 6 shows results related to clinical data. The mean and standard deviation of the estimated kinetic parameters for a different number of iterations, #iter, are shown; the standard deviation is relevant to the subjects' kinetic parameters variability. Parameters are evaluated from two ROIs covering the gray matter (results in Figure 6) and white matter (results in Figure 6).

**Figure 7:**
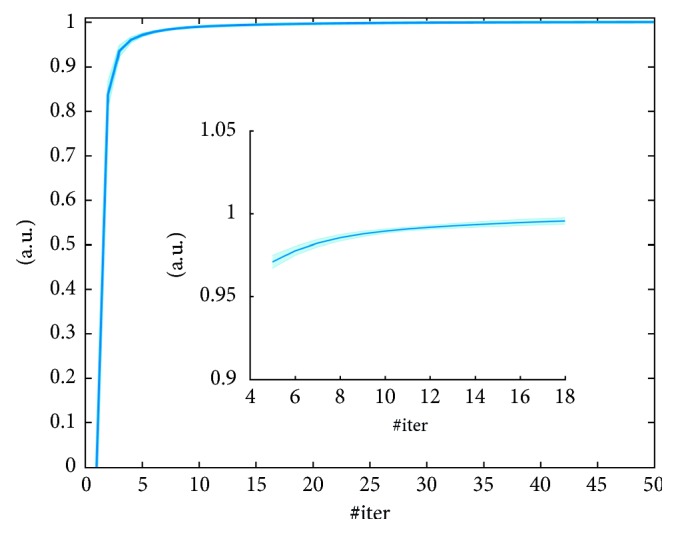
norm*L*_*n*_ of the ICM-EM algorithm evaluated on clinical data. Inner box: zoomed version of the graph.

In Figure 7, the mean and standard deviation of the norm*L*_*n*_ values are shown, as a function of the number of direct iterations; the standard deviation is relevant to the subjects' kinetic parameters variability. A zoom of the graph is also shown.

**Figure 8:**
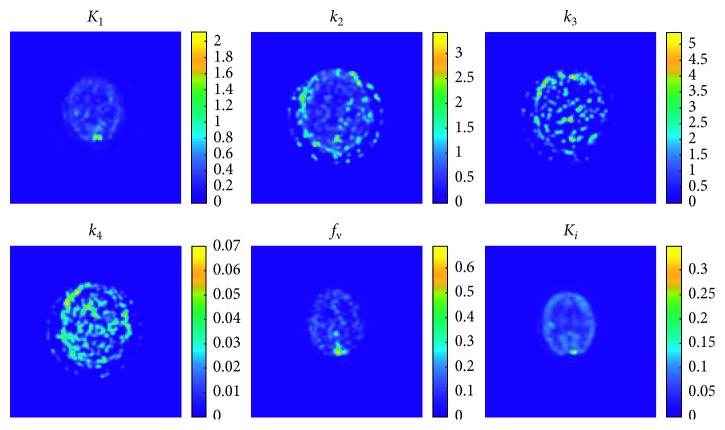
Parametric maps obtained from subject data and reconstructed with 10 direct iterations.

**Figure 9:**
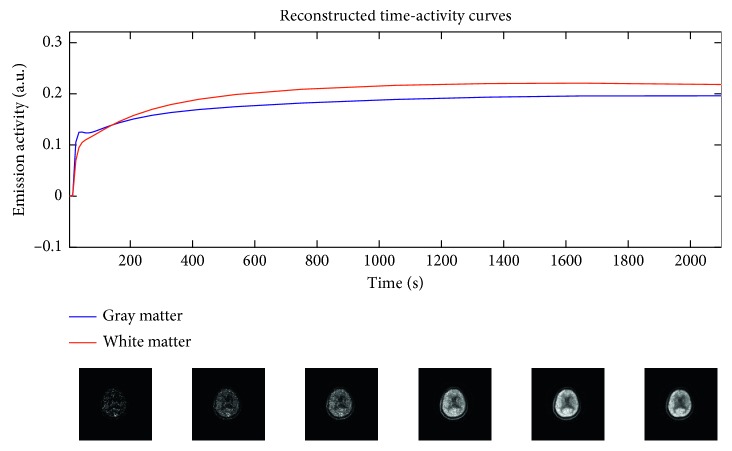
Dynamic reconstructed images obtained from subject data and reconstructed with ten direct iterations: (a) TACs reconstructed from the images; (b) reconstructed temporal images.

In Figure 8, as an example, the parametric maps obtained from a subject data and reconstructed with 10 direct iterations are shown. The reconstructed dynamic images are shown in Figure 9. Similarly to what shown in Figure 5 for simulation results, Figure 9 shows the TACs reconstructed from the images and Figure 9 shows six images relevant to six different time intervals.

## 4. Discussion

In the last few years, many groups have proposed different methods for estimating the parametric maps directly from the measured data [7, 11–15]. Some of them focused on the usage of linear kinetic models [17–19], whereas others developed methods tailored for nonlinear compartmental models [13, 22, 24]. Wang and Qi [25] proposed a generalized reconstruction method, which utilizes surrogate functions for optimization transfer expectation maximization (OTEM). This approach decouples the reconstruction problem from the nonlinear kinetic fitting, at each iteration; this allows using well-established least-squares optimization procedures.

In this paper, we present a new approach, based on a probabilistic modeling of the direct reconstruction problem and partly inspired by the OTEM method. Moreover, the proposed method exploits an analytical approach to NLS fitting of complex compartmental kinetic models, by means of a set of auxiliary parameters, reducing computational errors and approximations, and computing time.

Apart from the theoretical basis of its derivation, the present work has two main differences, compared with the original OTEM. One is the change from a kinetic model defined as a system of ordinary differential equations in terms of the kinetic constants [*K*_1_, *k*_2_, *k*_3_, *k*_4_] to an exponential form described with respect to a set of auxiliary variables [*α*_1_, *α*_2_, *β*_1_, *β*_2_] (12). The other one is the derivation of an analytical expression for the convolution operation between the blood input function and the tissue impulse response function, avoiding the need for time-consuming numerical approximations, giving the final model described in (16). These should lead to acceleration of the convergence rate; moreover, the combination of the Feng model with the exponential model resulted to bring computational benefits.

As mentioned in Section 2.1, our ICM-based reconstruction approach is transparent to the choice of the kinetic model used in (5), both in terms of its theoretical formulation and its actual implementation. For this reason, we thought it was out of the scope of the current work to in-depth investigation of the speed-up factor provided by the analytic formulation presented in (16); a separate work is planned for evaluating how it could lead, by itself, to a speed-up of about 100x when compared to conventional numeric implementation.

The use of the exponential formulation of the kinetic model is not new in the field of PET kinetic models, and its derivation is well described both in [3, 13]. However, its use in the context of direct reconstruction is not well documented. Kamasak et al. [13] used this convention in the definition of their direct reconstruction method, but the final model was restricted to work just for a specific class of the kinetic model, while both the OTEM algorithm and the proposed ICM-EM algorithm are designed to be flexible and adaptable to a wide range of different applications (i.e., different tracers, anatomic region, or kinetic model).

Simulation results allowed us to analyze the behavior of the proposed method in different noisy conditions to test its reliability even on noisy data. Moreover, by using simulation data, we can try to determine the optimal number of iterations to assess the kinetic parameter values.

The results shown in Figure 2 regarding simulated data analysis demonstrate that the ICM-EM algorithm gives a good estimation of the kinetic parameters at any noisy condition, for both the gray and the white matters; in fact, the MSE values are rather low: MSE is <10^−2^ for all the estimated parameters. From the comparison between Figure 2(a) and Figure 2(b) graphs, it results that the MSE from the white matter region is quite similar to the one from the gray matter. As far as the different noisy conditions, as expected, the MSE value is lower for lower noise conditions and it increases as the noise increases.

For what concerns the analysis of the minimum number of iterations to execute to obtain a good parameters estimation, from Figure 2 we can see that after a first transition phase, that ends at about 10 #iter, the MSE value does not undergo major changes.

Since we are using a nonlinear model with a high number of parameters to be estimated, it should be possible that the optimization ends up converging to local minima. We tried to remove this uncertainty by repeating simulations for different initial values of the parameters; the results obtained were each time very similar to the ones shown in Figure 2.

The normalized log-likelihood norm*L*_*n*_ evaluated on the simulated data, as it is shown in Figure 3, demonstrates that the ICM-EM algorithm has a good convergence rate at any noisy condition. Moreover, as it is better shown in the zoomed part of the graph, the convergence rate is lower in noisier conditions; it was an expected result. From Figure 3, we can deduce that about ten direct iterations can be sufficient for guaranteeing the convergence.

As an example of parametric maps and images reconstruction results, Figures 4 and 5 show the results obtained with ten direct iterations; from the resulting parametric and reconstructed images, it is evident that ten iterations seem to be enough for obtaining a good estimation of parameters and images reconstruction. Only the *k*_4_ parametric map appears quite noisy, not allowing to discern well the gray matter from the white matter; it is mainly due to the low values of the real *k*_4_, and to the small difference between the gray and white matter real *k*_4_ (Table 1). However, we need to take into account how, with a limited observation duration (40 minutes in the present study), parameters with very low values, as *k*_4_, are not actually observable. Our choice for the acquisition length was meant to mimic the protocol used in common clinic brain dynamic scans, while the choice of a very low but nonzero *k*_4_ value had the intention to model some uncertainty around the hypothesis of irreversible tracer conventionally used for the brain [18F]FDG kinetic modeling. Given a simulated dataset generated with a 4-*k* compartmental model, we decided to use the same model also in data fitting, being aware of the unreliability of the *k*_4_ map estimate.

In this work, we applied the ICM-EM algorithm on clinical data, coming from six normal subjects; it allowed us to perform a preliminary and simple statistics on resulting data for evaluating the goodness of the method.

The results shown in Figure 6 are relevant to the estimated kinetic parameters evaluated on clinical data for gray and white matter regions. From the figures, we can conclude that the estimated parameters values are quite consistent especially for the number of iterations greater than ten.

The standard deviation in the figures shows the kinetic parameters variability; the subjects enrolled in the study were diagnosed without brain pathologies, so we could suppose that the variability obtained was mainly due to the ‘biological' variability as well as to the estimation method.

As it was demonstrated on simulated data, also on clinical data (Figure 7), the normalized log-likelihood norm*L*_*n*_ at about ten iterations seems to have reached convergence.

The parametric maps shown in Figure 8 are obtained from a subject's data, reconstructed with 10 direct iterations. Differently from the ROIs analysis, the maps allow highlighting the geometric distribution of each kinetic parameter, giving an overall spatial vision of the tissue metabolic behavior; however, due to the point-to-point analysis of the kinetic curves, the maps result very noisy, especially for the microparameters (*K*_1_,…, *k*_4_, *f*_v_).

As previously discussed, *k*_4_ maps appear very noisy and with values very close to zero, for any anatomic region. This supports the assumption that a kinetic model with three rate parameters is more suitable to describe the behavior of the [18F]FDG tracer in the brain, and the nonobservability of too-low-valued parameters with a 40-minute scan. To be consistent with the simulation study and to be as general as possible in our testing, we decided to keep using a 4-*k* model for the clinical data sets, too: in this case, the negligible value of *k*_4_ should not be considered in the evaluation of metabolic behavior.

The focus of this work was to propose and test a 4D reconstruction method for direct parametric maps estimation, and its test on a small number clinical cases has to be seen just as a showcase of its behavior. A proper study of the quality of the estimated maps in a clinical setting, which is indeed more dependent on the correct choice of the model to which our method is transparent, should be the focus of a future work, based on a wider number of clinical cases.

The results shown in Figure 9 are relevant to the temporal behavior of the reconstructed images from a single subject data. The example reconstructed PET images shown for simulated (Figure 5) and clinical data (Figure 9) are obtained after ten iterations, as from the previous analysis it seems that 10 should be a sufficient number of iterations for estimating kinetic parameters with the ICM-EM algorithm. However, it is worth to note that a characteristic of the direct reconstruction algorithms, and in particular the ICM-EM algorithm just as it is conceived, is that the reconstruction noise no longer increases with the number of iterations, as it happens on conventional iterative reconstruction algorithms. So, increasing the number of iterations should not worsen the images quality and the only downside should be the computation time.

For [18F]FDG, the clinically relevant parameter is normally the macroparameter *K*_*i*_; this is usually estimated using linearized Patlak-based graphical methods [18, 19, 31, 32]. This is done especially because using linear models is much easier and robust. However, only macroparameter estimation may not be sufficient to describe all the details of the metabolic process under investigation.

On the other hand, microparameter estimate gives additional and detailed information about the phenomenon that is being studied. Our proposed method allows the estimation of microparameters and the derivation, from them, of the macroparameter *K*_*i*_. In this work, we did not perform the comparison of the *K*_*i*_ parameter estimated with our method and the Patlak-based methods because, in our view, it goes beyond the main purpose of this work.

## 5. Conclusion

In this work, we proposed and tested a direct parametric image reconstruction algorithm from dynamic PET data. This proposed method was tested on simulated and clinical data.

Working with simulations, we analyzed the behavior of the proposed method under different noisy conditions, to test its reliability even on noisy data; this analysis led to the conclusion that the ICM-EM algorithm gives a good estimation of the parametric maps in any noisy condition.

By analyzing simulation results, we also tried to determine the optimal number of iterations needed to assess the kinetic parameter values properly, and we ended up verifying that the algorithm can deal with almost every noisy condition in just about ten iterations.

We applied the ICM-EM algorithm to clinical data, coming from six normal subjects; the results obtained seem promising for further studies.

## Figures and Tables

**Table 1:** Kinetic parameter values used for the simulation.

	*K*_1_ (ml·min^−1^·g)	*k*_2_ (min^−1^)	*k*_3_ (min^−1^)	*k*_4_ (min^−1^)	*f*_v_ (%)
Gray matter	0.6805	0.3945	0.0533	0.0031	0.0985
White matter	0.4091	0.3276	0.0451	0.0015	0.1160

## References

[1] Dimitrakopoulou-Strauss A., Strauss L. G., Heichel T. (2002). The role of quantitative (18)F-FDG PET studies for the differentiation of malignant and benign bone lesions. *Journal of Nuclear Medicine*.

[2] Schmidt K. C., Turkheimer F. E. (2002). Kinetic modeling in positron emission tomography. *Quarterly Journal of Nuclear Medicine*.

[3] Gunn R. N., Gunn S. R., Cunningham V. J. (2001). Positron emission tomography compartmental models. *Journal of Cerebral Blood Flow & Metabolism*.

[4] Zhou Y., Huang S. C., Bergsneider M. (2001). Linear ridge regression with spatial constraint for generation of parametric images in dynamic positron emission tomography studies. *IEEE Transactions on Nuclear Science*.

[5] Kamasak M. E. (2014). Effects of spatial regularization on kinetic parameter estimation for dynamic PET. *Biomedical Signal Processing and Control*.

[6] Loeb R., Navab N., Ziegler S. I. (2015). Direct parametric reconstruction using anatomical regularization for simultaneous PET/MRI data. *IEEE Transactions on Medical Imaging*.

[7] Reader A. J., Verhaeghe J. (2014). 4D image reconstruction for emission tomography. *Physics in Medicine and Biology*.

[8] Qi J., Leahy R. M. (2006). Iterative reconstruction techniques in emission computed tomography. *Physics in Medicine and Biology*.

[9] Santarelli M. F., Della Latta D., Scipioni M., Positano V., Landini L. (2016). A Conway–Maxwell–Poisson (CMP) model to address data dispersion on positron emission tomography. *Computers in Biology and Medicine*.

[10] Santarelli M. F., Positano V., Landini L. (2017). Measured PET data characterization with the negative binomial distribution model. *Journal of Medical and Biological Engineering*.

[11] Wang G., Qi J. (2013). Direct estimation of kinetic parametric images for dynamic PET. *Theranostics*.

[12] Tsoumpas C., Turkheimer F. E., Thielemans K. (2008). A survey of approaches for direct parametric image reconstruction in emission tomography. *Medical Physics*.

[13] Kamasak M. E., Bouman C. A., Morris E. D., Sauer K. (2005). Direct reconstruction of kinetic parameter images from dynamic PET data. *IEEE Transactions on Medical Imaging*.

[14] Tsoumpas C., Turkheimer F. E., Thielemans K. (2008). Study of direct and indirect parametric estimation methods of linear models in dynamic positron emission tomography. *Medical Physics*.

[15] Rahmim A., Tang J., Zaidi H. (2009). Four-dimensional image reconstruction strategies in dynamic PET: beyond conventional independent frame re-construction. *Medical Physics*.

[16] Besag J. E. (1986). On the statistical analysis of dirty pictures. *Journal of the Royal Statistical Society: Series B*.

[17] Meikle S. R., Matthews J. C., Cunningham V. J. (1998). Parametric image reconstruction using spectral analysis of PET projection data. *Physics in Medicine and Biology*.

[18] Angelis G. I., Thielemans K., Tziortzi A. C., Turkheimer F. E., Tsoumpas C. (2011). Convergence optimization of parametric MLEM reconstruction for estimation of Patlak plot parameters. *Computerized Medical Imaging and Graphics*.

[19] Wang G., Fu L., Qi J. (2008). Maximum a posteriori reconstruction of the Patlak parametric image from sinograms in dynamic PET. *Physics in Medicine and Biology*.

[20] Leahy R. M., Qi J. Y. (2000). Statistical approaches in quantitative positron emission tomography. *Statistics and Computing*.

[21] Ollinger J. M., Fessler J. A. (1997). Positron-emission tomography. *IEEE Signal Processing Magazine*.

[22] Matthews J. C., Angelis G. I., Kotasidis F. A., Markiewicz P. J., Reader A. J. Direct reconstruction of parametric images using any spatiotemporal 4D image based model and maximum likelihood expectation maximization.

[23] Marquardt D. W. (1963). An algorithm for least-squares estimation of nonlinear parameters. *Journal of the Society for Industrial and Applied Mathematics*.

[24] Wang G., Qi J. (2010). Acceleration of the direct reconstruction of linear parametric images using nested algorithms. *Physics in Medicine and Biology*.

[25] Wang G., Qi J. (2012). An optimization transfer algorithm for nonlinear parametric image reconstruction from dynamic PET data. *IEEE Transactions on Medical Imaging*.

[26] Feng D., Huang S. C., Wang X. (1993). Models for computer simulation studies of input functions for tracer kinetic modeling with positron emission tomography. *International Journal of Bio-Medical Computing*.

[27] Smith B. R., Hamarneh G., Saad A. Fast GPU fitting of kinetic models for dynamic PET.

[28] Fessler J. *Image Reconstruction Toolbox*.

[29] Zubal I. G., Harrell C. R., Smith E. O., Rattner Z., Gindi G., Hoffer P. B. (1994). Computerized three-dimensional segmented human anatomy. *Medical Physics*.

[30] Cherry S. R., Sorenson J. A., Phelps M. E. (2012). *Physics in Nuclear Medicine*.

[31] Wang G., Qi J. (2009). Generalized algorithms for direct reconstruction of parametric images from dynamic PET data. *IEEE Transactions on Medical Imaging*.

[32] Patlak C. S., Blasberg R. G. (1985). Graphical evaluation of blood-to-brain transfer constants from multiple-time uptake data. Generalizations. *Journal of Cerebral Blood Flow and Metabolism*.

